# Lifestyle factors and visceral adipose tissue: Results from the PREDIMED-PLUS study

**DOI:** 10.1371/journal.pone.0210726

**Published:** 2019-01-25

**Authors:** Aina M. Galmes-Panades, Jadwiga Konieczna, Itziar Abete, Antoni Colom, Núria Rosique-Esteban, Maria Angeles Zulet, Zenaida Vázquez, Ramón Estruch, Josep Vidal, Estefanía Toledo, Nancy Babio, Miguel Fiol, Rosa Casas, Josep Vera, Pilar Buil-Cosiales, José Antonio de Paz, Albert Goday, Jordi Salas-Salvadó, J. Alfredo Martínez, Dora Romaguera, on behalf of PREDIMED-Plus investigators

**Affiliations:** 1 Balearic Islands Health Research Institute (IdISBa), University Hospital Son Espases, Palma de Mallorca, Spain; 2 CIBER Pathophysiology of Obesity and Nutrition (CIBEROBN), Health Institute Carlos III (ISCIII), Madrid, Spain; 3 Department of Nutrition, Food Sciences and Physiology, Center for Nutrition Research, University of Navarra (UNAV), Pamplona, Spain; 4 Human Nutrition Unit, University Hospital of Sant Joan de Reus, Department of Biochemistry and Biotechnology, Pere Virgili Institute for Health Research, Rovira i Virgili University, Reus, Spain; 5 Department of Preventive Medicine and Public Health, University of Navarra, Pamplona, Spain; 6 Department of Internal Medicine, IDIBAPS, Hospital Clinic, University of Barcelona, Barcelona, Spain; 7 Department of Endocrinology, IDIBAPS, Hospital Clinic, University of Barcelona, Barcelona, Spain; 8 CIBER Diabetes and Metabolic Diseases (CIBERdem), Health Institute Carlos III (ISCIII), Madrid, Spain; 9 Institute for Biomedical Research August Pi i Sunyer (IDIBAPS), Barcelona, Spain; 10 Primary Care, Health Service of Navarra-Osasunbidea, Pamplona, Spain; 11 Division of Preventive Medicine, University of León, León, Spain; 12 Lipids and Cardiovascular Epidemiology Research Unit, Municipal Institute of Medical Research (IMIM), Barcelona, Spain; 13 Madrid Institute for Advanced Studies (IMDEA) Food Institute, Madrid, Spain; University of Cordoba, SPAIN

## Abstract

**Background:**

Visceral adipose tissue (VAT) is a strong predictor of cardiometabolic health, and lifestyle factors may have a positive influence on VAT depot. This study aimed to assess the cross-sectional associations between baseline levels of physical activity (PA), sedentary behaviours (SB) and adherence to the Mediterranean diet (MedDiet) with VAT depot in older individuals with overweight/obesity and metabolic syndrome.

**Methods:**

Baseline data of the PREDIMED-Plus study including a sample of 1,231 Caucasian men and women aged 55–75 years were used. Levels of leisure-time PA (total, light, and moderate-to-vigorous, in METs·min/day) and SB (total and TV-viewing, in h/day) were evaluated using validated questionnaires. Adherence to the MedDiet was evaluated using a 17-item energy-restricted MedDiet (erMedDiet) screener. The chair-stand test was used to estimate the muscle strength. VAT depot was assessed with DXA-CoreScan. Multivariable adjusted linear regression models were used to evaluate the association between lifestyle factors and VAT. For the statistics we had used multiadjusted linear regression models.

**Results:**

Total leisure-time PA (100 METs·min/day: β -24.3g, -36.7;-11.9g), moderate-to-vigorous PA (β -27.8g, 95% CI -40.8;-14.8g), chair-stand test (repeat: β -11.5g, 95% CI -20.1;-2.93g) were inversely associated, and total SB (h/day: β 38.2g, 95% CI 14.7;61.7) positively associated with VAT. Light PA, TV-viewing time and adherence to an erMedDiet were not significantly associated with VAT.

**Conclusions:**

In older adults with overweigh/obesity and metabolic syndrome, greater PA, muscle strength, and lower total SB were associated with less VAT depot. In this study, adherence to an erMedDiet was not associated with lower VAT.

## Introduction

Excess of visceral adipose tissue (VAT), which appears with increasing age, has been shown to be associated with cardiovascular disease (CVD), type 2 diabetes (T2D), and all cause-mortality, beyond general obesity [1]. The evaluation of how modifiable lifestyle factors such as diet and physical activity (PA) influence VAT depot, may help to develop preventive strategies to combat the morbimortality associated to obesity.

There is growing evidence supporting a negative association between aerobic PA and VAT, suggesting that, for VAT reduction, the intensity and volume of PA appears to be especially important [2–5]. In this regard, moderate-to-vigorous PA (MVPA) has been shown to exert greater effect on VAT than activities at lower intensities [2,6], albeit the role of low intensity PA, as well as the exact influence of exercise volume on VAT, has not been clearly established yet [7]. Additionally, sedentary behaviours (SB), have been associated with the increment of VAT in some studies [3,8] but not in others [4,9]. Moreover, previously published cross-sectional results from the PREDIMED-Plus trial showed that MVPA and SB were associated in opposite directions with the prevalence of abdominal obesity, determined by waist circumference (WC) [10]; hence, more detailed investigation of the association between PA and SB with VAT is warranted, particularly given that studies in this field are typically hampered by small sample size.

Not only PA or SB, but also lifestyle changes targeting dietary patterns—and particularly the Mediterranean diet (MedDiet)—has been associated with lower abdominal fat accumulation [11–13]. Moreover, it has been suggested that some of the effects of this dietary pattern on the incidence of chronic diseases are mediated by its effect on the accumulation of VAT [14]. In this sense, results from the recent PREDIMED trial have shown that intervention with MedDiet may counteract the harmful effects of increased abdominal adiposity on the risk of CVD [15]. However, previous studies addressing this issue were based on the anthropometric measurements of WC, as proxy indicator of visceral fat. Future studies with precise imaging-defined measures of VAT are needed to elucidate whether MedDiet is able to exert favorable changes in this pathogenic fat deposition.

Based on aforementioned considerations, the aim of this study was to evaluate the cross-sectional associations between lifestyle factors, i.e. time spend in different intensity levels of PA, SB and adherence to MedDiet with VAT depot, assessed accurately by dual-energy X-ray absorptiometry (DXA). The association between VAT and objectively measured indicator of muscle strength was also addressed, in order to support the analysis with self-reported PA and SB.

## Material and methods

### Study overview and sample

The PREDIMED-Plus study, is a 6-year ongoing multicenter, randomized, parallel-group, primary prevention, clinical trial conducted in Spain to assess the effect of a weight loss intervention program based on an energy-restricted traditional MedDiet (erMedDiet), PA promotion and behavioral support, in comparison with an usual care intervention only with energy-unrestricted Mediterranean diet without any advice to increase PA or losing weight (control group, CG) on hard composite of CVD events (cardiovascular death, nonfatal myocardial infarction, and nonfatal stroke), as a primary end point. The intervention group (IG) received personalized recommendations during face-to-face individual sessions with dieticians (1/month during the first year and 1/3 months afterwards), in order to increase progressively their levels of PA (to at least 150 minutes/week of moderate-to-vigorous intensity), an adherence to an erMedDiet and behavioral support. In addition to the individual meetings, all participants received group sessions (and telephone calls) 1/month in the IG and 1/6 months in the CG, to provide informative talks addressing healthy lifestyle, during which free extra-virgin olive oil (1 L/month) and raw nuts (125 g/month) were provided in both groups. The intervention will last for 6 years, with a further 2 years of follow-up for collection of clinical events.

Of a total of 9677 individuals assessed for eligibility during the 4-week run-in period, a sample of 6874 participants were recruited and randomized between 2013 and 2016 in 23 Spanish centers. Eligible participants were men aged 55–75 and women aged 60–75 years, with BMI ≥ 27 and < 40 kg/m^2^, who met ≥ 3 components of the metabolic syndrome (MetS) [16]. All participants provided written informed consent. The study protocol was approved by the Research Ethics Committees from all recruiting centers according to the ethical standards of the Declaration of Helsinki: CEI de la Universidad de Navarra, CEIC de León, CEI de las Illes Balears, CEIC del Hospital Clínic de Barcelona, CEIC del Hospital Universitari Sant Joan de Reus. The trial was registered at the International Standard Randomized Controlled Trial (ISRCTN: http://www.isrctn.com/ISRCTN89898870) with number 89898870 and registration date of 24 July 2014. Details of the study protocol have been described elsewhere [10,17] and are available on the website http://www.predimedplus.com/.

Of the total PREDIMED-Plus population, a subsample of 1532 participants coming from 7 recruiting centers with DXA scanner available, underwent total body DXA scans at baseline. Of those, 1289 participants from 6 centers had data on VAT within the android region, as these centers had access to the latest software to derive VAT from the DXA scans (CoreScan). Participants with no data available on any of the exposure variables or the covariables, and whose DXA measurements exceeded planned time of DXA exploration were excluded from the analyses (n = 58). Finally, a number of 1231 participants was included in the present analysis (see Flow chart in S1 Fig). We used the study baseline database generated in August 2017.

### Exposure assessment

Total leisure-time PA was assessed using the validated self-reported REGICOR questionnaire [18], which includes questions to collect information about the type of activity, frequency (number of days) and duration (min/day). An intensity code was assigned to each activity based on the compendium of PA in Metabolic Equivalent Tasks (METs) [19]. The information about 6 types of activities performed during a conventional month (excluding special situations like holidays or periods of sick leave) was collected by trained interviewer and categorized into three PA intensity levels: light (*<*4 MET)—walking at a slow/normal pace; moderate (4–5.5 MET)—brisk walking, working in the garden; and vigorous (≥6.0 MET)—walking in the countryside, climbing stairs, exercise or play sports at home, outdoors or in a gym. Total PA was estimated as the summed product of frequency, duration and intensity of each activity divided by 30 days/month (MET·min/day), and MVPA was calculated as the sum of moderate and vigorous PA.

Time spent on SB, defined as any behavior conducted awake with an energy cost ≤1.5 MET while in a sitting or reclining posture [20], was evaluated on weekdays and weekends with the validated Nurses’ Health Study questionnaire [21]. The questionnaire consisted of a four of open-ended questions assessing the average daily time spent over the last year in watching TV, sitting while using computer, sitting on journeys (for work purposes or leisure time, as driver or passenger car, subway, bus, etc) and total sitting (counting any time spend sitting). Answers included 12 categories ranging from 0 to 9 h/day of sitting time for the corresponding activity. Time spent on the total SB (h/day) (calculated as [time during each weekday*5 + time during each weekend day*2]/7) and on the TV-viewing SB, proposed as the most prevalent form of time spent on SB among aged population [22], were used for the present study.

We also used data on objectively measured muscle strength, as a supportive analysis for the association between PA and SB with VAT. Lower-limb muscle strength/physical fitness was determined at baseline using previously validated in community-dwelling older subjects 30-s chair-stand test [23]. This test consists on counting the number of stand-sit on a chair cycles within 30 seconds.

Baseline adherence to an erMedDiet was assessed using of the 17-point screening questionnaire collecting data with reference to the last year [17](S1 Table). This is the modified version of 14-item dietary questionnaire previously validated in Spanish population [24]. A value of 1 in case of meeting the specific criteria or 0 otherwise, was recorded for each item. The final score ranged from 0 to 17 was developed; the higher the score, the better adherence to the MedDiet.

For the sensitivity analysis, another Mediterranean Diet Score (MDS) was constructed as described by Trichopoulou *et al*. [25], considering the consumption of nine food groups or nutrients (cereals, fruits & nuts, vegetables, legumes, fish, meat, dairy products, ratio of mono-unsaturated to saturated fatty acids and alcohol), based on the data collected in previously validated 143-item semi-quantitative food frequency questionnaire (FFQ) referred to food intake over last year [26]. Each food or nutrient was scored with 1 point if consumption was in line with the Mediterranean diet, and with 0 points otherwise. The final score ranging from 0 to 9 points was developed by summing the 9 foods; the higher the score, the better adherence to the MedDiet.

### Outcome assessment

Baseline data on total and regional body composition (fat, lean and bone mass) was measured using 2 types of DXA equipment belonging to the third-generation scanners from GE (DXA Lunar Prodigy Primo (2 centers) and Lunar iDXA (4 centers); GE Healthcare, Madison, WI) connected with enCore^™^ software. Among different regions of interest, the abdominal android region was defined, using the software provided by the manufacturer, as the area that begins at the top of the iliac crest toward the head for 20% of the distance from the iliac crest to the base of the skull. Within the android region, we focused on VAT, as we previously found in the same sample of subjects that it has better capacity than anthropometric and other DXA-related parameters (total body fat, fat in trunk or android region) in prediction of cardiometabolic risk [27]. For VAT measures, scans were reanalyzed using validated CoreScan software application [28], which algorithms work through detection of the width of the subcutaneous (SAT) layer on the lateral part of the abdomen and the anterior–posterior thickness of the abdomen, by X-ray attenuation of the abdominal cavity in the android region. This is automated procedure developed by the manufacturer. DXA scans were performed by trained operators following standard protocol and subject positioning provided by the manufacturer. Participants were scanned wearing examination gown. The DXA was phantom calibrated daily according to manufacturer guidelines.

### Covariable assessment

Baseline data on sex, age, smoking habits, educational level, marital status, medical conditions and medication use have been evaluated using self-reported questionnaires on socio-demographic factors. Body weight (kg) and height (cm) were measured in light clothing and without shoes with use of calibrated scale and a wall-mounted stadiometer, respectively. Blood samples collected after overnight fast were used to performed biochemical analyses, such as glycated hemoglobin (HbA1c, %) using standard routine methods. Previously validated FFQ [26] was used to estimate baseline information on total energy intake (kcal/day), alcohol intake (g/day) and fatty acids (g/day). Smoking habits was categorized as current, former and never smoker, educational level was categorized as higher education/technician, secondary education, illiterate/primary education, and marital status was categorized as married and single/separated/divorced/widow(er). Dichotomous variable (yes/no) was generated for diabetes status, defined as diagnosed diabetes self-reported at inclusion or baseline HbA1c ≥ 6.5% or use of antidiabetic medication at baseline, such as insulin, metformin (in case of diagnosed diabetes or Hba1c ≥ 6.5%) or others.

### Statistical analysis

Data are expressed as means and standard deviations (SDs) for continuous variables or numbers and percentages (%) for categorical variables. One-way analysis of variance (ANOVA) and chi-square tests (χ^2^) (for continuous and categorical variables, respectively) were used to evaluate difference in baseline characteristics of the study participants between sample taken to the analysis and total sample randomized for the trial.

First generalized additive models were applied to ascertain about the linearity in the association between our exposures and the outcome. Given that there was no evidence of departure from linearly, multivariate linear regression analyses were used to estimate the β-coefficients and 95% confidence intervals (CI) for the associations between our exposures as continuous variables (PA per 100 MET·min/day, SB in h/day, chair-stand test in number of repeats, and erMedDiet score per point increment) with our outcome, VAT (g). We run Model 1 adjusting for age, sex, and recruiting center. Model 2, was adjusted by the minimally sufficient adjustment set, determined using Directed Acyclic Graphs (DAGs) implemented in DAGitty software [29] available free on www.dagitty.net. The DAGs were built by identifying all known factors affecting each of our exposures on VAT (see DAGs in S2 Fig). Thus, the covariables used in Model 2 included age, sex, recruiting center, erMedDiet score (for models with exposures PA, chair-stand test and SB), alcohol (PA, chair-stand test), smoking habits (PA, chair-stand test, erMedDiet), educational level (SB), and total PA (erMedDiet). In addition, height was added to Model 2 as adjusted variable to account for differences in body dimensions. A last Model 3 was also build to evaluate the independent associations of exposure variables by mutually adjusting one for each other (models on PA and chair-stand test were further adjusted for total SB; models on SB were further adjusted for total PA; and models on erMedDiet were adjusted for SB and for total PA).

We further analyzed the associations on VAT depot, according to the compliance of the World Health Organization (WHO) recommendations for adults on MVPA (at least 150 min/week of MVPA and for more benefits on health, at least, 300 min/week of MVPA) [30]. For that, we categorized MVPA (in min/week) into three categories: <150 min/week; 150–300 min/week; and ≥300 min/week, and we used this categorized exposure variable in a lineal regression model adjusted as previously indicated in Model 2 for PA. For SB, we defined three categories according to the daily hours spent in any sedentary activity: high sedentarism (≥7 h/day), low sedentarism (<4 h/day) and intermediate category (4–7 h/day). These cut-off points were based on findings from previous publications [21,31]. The association between categories of SB and VAT were evaluated using linear regression adjusted as in Model 2. We also examined the joint association of WHO recommendations on MVPA and SB categories with VAT depot.

We performed several sensitivity analyses: possible effect modifications by sex, age in two categories (using as cut-off the sex-specific median: men 64 and women 67 years), obesity (yes or no, defined as a BMI ≥30 kg/m^2^) and diabetes status (diabetic, non-diabetic) were also evaluated by adding cross-product terms between these variables and our exposure variables to the linear regression models. Moreover, we evaluated the association between the MDS and VAT using linear regression models 1 and 2. Finally we evaluated how further adjustment for DXA-measured total body fat (%) affected the associations between our exposures and VAT.

Statistical analyses were performed using Stata v15.0 program.

## Results

Table 1 presents comparison of participants’ characteristics at baseline between sample taken to the analysis and total sample randomized for the trial. In general terms, the sample used in our analysis was to a large degree representative of the total sample from PREDIMED-Plus trial. For some characteristics, the difference between samples reached statistical significance, although the magnitude of the difference was low. S2 Table shows the same participants´ characteristics for study sample by recruiting center.

**Table 1:** Baseline characteristics of the total study population and the sample used for analysis.

	Total sample n = 6874	Subsample n = 1231	*p*-*value*
Age (years)	64.9 (4.9)	65.3 (5.0)	**0.003**
Sex:			
Men	3539 (51)	647 (53)	0.405
Women	3335 (49)	584 (47)	
BMI (kg/m^2^)	32.6 (3.5)	32.5 (3.3)	0.867
Height (cm)	163 (9)	163 (9)	0.874
VAT (kg)		2.29 (0.89)	
Total fat (kg)		34.4 (7.2)	
MetS components prevalence:			
Abdominal obesity	6607 (96)	1187 (96)	0.535
Hyperglycemia	5178 (75)	918 (75)	0.498
Hypertriglyceridemia	3824 (56)	675 (55)	0.535
Low HDL-cholesterol	2947 (43)	564 (46)	**0.021**
Hypertension	6308 (92)	1145 (93)	0.079
Total PA (MET·min/day)	359 (339)	396 (338)	**<0.001**
MVPA (MET·min/day)	250 (320)	282 (322)	**<0.001**
Light PA (MET·min/day)	109 (136)	114 (136)	0.127
Chair-stand test (repeats)	13.2 (5.1)	13.9 (5.3)	**<0.001**
Total SB (h/day)	6.01 (1.96)	5.80 (1.81)	**<0.001**
TV-viewing SB (h/day)	3.26 (1.72)	3.10 (1.62)	**<0.001**
erMedDiet score (points)	8.50 (2.68)	8.31 (2.63)	**0.010**
MDS (points)	4.35 (1.63)	4.37 (1.65)	**<0.001**
Alcohol (g/day)	11.2 (15.3)	11.3 (14.9)	0.649
Smoking habits:			0.369
Never	3006 (44)	527 (43)	
Current	857 (12)	146 (12)	
Former	2983 (44)	558 (45)	
Educational level:			0.771
Higher education/technician	1521 (22)	268 (22)	
Secondary education	1984 (29)	368 (30)	
Primary education/illiterate	3302 (49)	595 (48)	

Data are expressed as means and standard deviations (SDs) for continuous variables or numbers and percentages (%) for categorical variables. Comparison between subsample (n = 1231) *versus* the remaining number of participants from total cohort (n = 5643) was determined using one-way analysis of variance (ANOVA) for continuous variables and chi-square test (χ^2^) for categorical variables. *P*-value threshold was set at <0.05.

MetS components, as one of the major inclusion criteria were evaluated at the first screening visit during run-in period, according to the harmonized definition of the joint statement from the International Diabetes Federation/National Heart, Lung and Blood Institute/American Heart Association (2009) [16].

Abbreviations: erMedDiet—energy-restricted Mediterranean diet; MetS—metabolic syndrome; PA—physical activity; MVPA—moderate-to-vigorous physical activity; SB—sedentary behaviours, VAT—visceral adipose tissue.

Table 2 shows the β-coefficients (95% CIs) of associations between reported PA, muscle strength measured by chair-stand test, SB and MedDiet score with VAT. In both minimally adjusted (Model 1) and multiple adjusted (Model 2) models, total PA and MVPA (100 MET·min/day), were inversely associated with VAT (estimates in Model 2: β -24.3g, 95% CI -36.7;-11.9g and β -27.8g, 95% CI -40.8;-14.8g, respectively, p<0.001). Furthermore, total SB (h/day) was positively associated with VAT (β 38.2g, 95% CI 14.7;61.7g, p = 0.001). Similarly, muscle strength, measured with chair-stand test (repeat) was associated inversely with VAT (β -11.5g, 95% CI -20.1;-2.93g, p = 0.009). After mutually adjustment for each other exposure, associations were slightly attenuated but remained statistically significant. No statistically significant associations between light PA, erMedDiet score, and TV-viewing on VAT were observed (p>0.05).

**Table 2:** Association of physical activity, sedentary behaviors and Mediterranean diet with visceral adipose tissue depots.

(n = 1231)	Model 1			Model 2			Model 3		
	β	(95% CI)	*p-value*	β	(95% CI)	*p-value*	β	(95% CI)	*p-value*
Total PA (100 MET·min/day)	-26.2	(-38.6; -13.7)	**<0.001**	-24.3	(-36.7; -11.9)	**<0.001**	-21.3	(-33.8; -8.78)	**0.001**
Light PA (100 MET·min/day)	4.92	(-26.2; 36.0)	0.757	5.05	(-25.5; 35.6)	0.746	7.33	(-23.1; 37.8)	0.637
MVPA (100 MET·min/day)	-29.6	(-42.7; -16.6)	**<0.001**	-27.8	(-40.8; -14.8)	**<0.001**	-24.8	(-37.9; -11.7)	**<0.001**
Chair-stand test (repeats)	-15.0	(-23.6; -6.33)	**0.001**	-11.5	(-20.1; -2.93)	**0.009**	-10.8	(-19.4; -2.21)	**0.014**
TV-viewing SB (h/day)	16.8	(-9.82; 43.4)	0.216	15.5	(-11.8; 42.8)	0.265	11.1	(-16.2; 38.4)	0.424
Total SB (h/day)	45.0	(21.8; 68.3)	**<0.001**	38.2	(14.7; 61.7)	**0.001**	31.4	(7.71; 55.1)	**0.009**
erMedDiet score (points)	-14.6	(-31.0; 1.86)	0.082	-10.4	(-26.7; 6.00)	0.215	-7.16	(-23.7; 9.34)	0.395

Abbreviations: CI—confidence intervals; erMedDiet—energy-restricted Mediterranean diet; MVPA—moderate-to-vigorous physical activity; PA—physical activity; SB—sedentary behaviours, VAT—visceral adipose tissue.

Model 1: Linear regression model adjusted for age, sex and center.

Model 2: Linear regression model adjusted for age, sex, center, erMedDiet score (for models with exposures total PA, light PA, MVPA, chair-stand test, total SB and TV-viewing SB), alcohol (total PA, light PA, MVPA, chair-stand test), smoking habits (total PA, light PA, MVPA, chair-stand test, erMedDiet score), educational level (total SB and TV-viewing SB), and total PA (erMedDiet score), and height (all exposures).

Model 3: Linear regression model 2, further adjusted for total SB (in models of total PA, light PA, MVPA chair-stand test and erMedDiet score) or total PA (in model of total SB, TV-viewing SB and erMedDiet score).

Table 3 shows the β-coefficients (95% CIs) of associations between meeting the WHO recommendations on MVPA and being more or less sedentary with VAT. Compared to those not meeting the WHO recommendations (MVPA <150 min/week), those participants dedicating between 150 and 300 min/week to MVPA showed -218 g lower VAT (95% CI -332;-104g), and those spending ≥300 min/week of MVPA showed -267 g lower VAT (95% CI -358;-175g), all *p*<0.001. Similarly, compared to those with very sedentary lifestyle (≥7 h/day), those dedicating 4–7 h/day to sedentary activities showed -60 g lower VAT (95% CI -153; 33g, *p* = 0.204) and those that were less sedentary (<4 h/day) showed -237 g lower VAT depot (95% CI -376;-97g, *p =* 0.001). Comparison of the joint association of both meeting the WHO recommendations on MVPA and sedentarism revealed that the lowest VAT depot was observed for those subjects that were less sedentary (<4 h/day) and devoted 150–300 min/week (β -478g, 95% CI -761;-195g) or ≥300 min/week (β- 391g, 95% CI -585;-197g) to MVPA.

**Table 3:** Association between adherence to the recommendations on physical activity according to WHO and sedentarism with visceral adipose tissue (n = 1231).

Variable	Categories	n	Mean (SD)	β (95% CI)	*p-value*
MVPA (min/week)^1^	<150	534	2354 (940)	Reference	
	150–300	219	2210 (851)	-218 (-332; -104)	**<0.001**
	>300	478	2263 (856)	-267 (-358; -175)	**<0.001**
Total SB (h/day)^2^	≥7	388	2399 (926)	Reference	
	4–7	686	2274 (891)	-60 (-153; 33)	0.204
	<4	157	2115 (789)	-237 (-376; -97)	**0.001**
Joint association MVPA (min/week) & Total SB (h/day)^3^	MVPA <150 & Total SB ≥ 7	205	2467 (973)	Reference	
	MVPA <150 & Total SB 4–7	278	2312 (941)	-34 (-165; 96.8)	0.610
	MVPA <150 & Total SB <4	51	2127 (734)	-323 (-544; -101)	**0.004**
	MVPA 150–300 & Total SB ≥ 7	79	2296 (807)	-231 (-417; -43.7)	**0.016**
	MVPA 150–300 & Total SB 4–7	111	2188 (903)	-241 (-409; -73.1)	**0.005**
	MVPA 150–300 & Total SB <4	29	2058 (759)	-478 (-761; -195)	**0.001**
	MVPA >300 & Total SB ≥ 7	104	2343 (913)	-271 (-442; -99.3)	**0.002**
	MVPA >300 & Total SB 4–7	297	2271 (837)	-318 (-449; -187)	**<0.001**
	MVPA >300 & Total SB <4	77	2128 (841)	-391 (-585; -197)	**<0.001**

Abbreviations: MVPA—moderate-to-vigorous physical activity; WHO—World Health Organization; SB—Sedentary Behaviour

^1^Levels of VAT (g) associated with adherence to the WHO recommendations on MVPA for adults (at least 150 min/week of MVPA and for more benefits on health, at least, 300 min/week of MVPA) [30] classified into three categories: <150 min/week; 150–300; and ≥300 min/week.

^2^Levels of VAT (g) associated with a time spent on total SB categorized as high (≥7 h/day), intermediate (4–7 h/day) and low (<4 h/day) according to findings from previous publications [21, 31].

^3^Levels of VAT (g) associated with joint adherence to MVPA and total SB

Association were determined by a linear regression models adjusted for age, sex, center, erMedDiet score, height, as well as smoking status and alcohol (in case of MVPA), and educational level (in case of sedentarism), and smoking status, alcohol and educational level (in case of joint adherence to MVPA and total SB).

Non-significant (*p*>0.05) interactions between our exposure variables and sex, age, obesity or diabetes on VAT depot were found; therefore stratified analysis by these variables was not conducted. We evaluated the association between another version of the MDS (score range 0–9) and results were similar to those of the erMedDiet score: in Model 1, one-point increment in the MDS was associated with -25.7 g (95% CI -51.7;0.24g, *p* = 0.052) less VAT and in Model 2, the estimate was -17.7 g (95% CI -43.5;8.00g, *p* = 0.176). Finally, further adjustment for total body fat to estimate the direct effect of our exposures on VAT (taking into account plausible mediators, according to our DAG) resulted in an attenuation of the effect estimates and loss of statistical significance (S3 Table).

## Discussion

It is well accepted that an excessive VAT accumulation, which appears while aging, carries associated metabolic disturbances, and increased mortality risk. Therefore, identification and development of strategies to prevent or decrease VAT is of high importance. Yet, it is unclear the contribution of lifestyle factors toward VAT modification, as most of the studies addressing this issue are severely hampered by small sample size or lack of accurate methods for VAT measurement. Thus, we used here DXA imaging technique to precisely measure visceral fat in the large cohort of older adults with overweight/obesity and MetS. Among the lifestyle factors studied, we found that total and MVPA (but not light PA), as well as total SB (but not TV-viewing SB) were associated with VAT depot (opposite directions). In this cross-sectional study, adherence to MedDiet was not significantly related to this pathogenic fat depot. Results of this study on the negative association between total PA, in terms of total energy expenditure, and imaging defined VAT depot corroborate most of previous cross-sectional [3,32,33] and interventional studies [6,34], conducted among different populations of adults, using self-reported [33,35,36] and accelerometry-derived PA data [3,32].

Mounting evidence point out the greater benefits of moderate-to-vigorous PA over light PA on health-related changes in adults (≥65 years or less), including lower rates of all-cause mortality, cardiorespiratory and metabolic diseases, osteoporosis, some cancers and depression [37]. Furthermore, elderly individuals may be unable to perform PA at higher intensity, giving a reason to focus on the role of light PA. The present analysis of PA intensities suggests that MVPA is favorable for VAT reduction in older overweight/obese subjects, while light PA has no significant association with this pathogenic depot. Our findings, although based on the self-reported data, are in line with other large cross-sectional studies performed in middle-age to elderly populations using both accelerometry and imaging techniques [3,4,32]. These observational findings have been supported in different exercise regimes. In a meta-analysis, Vissers *et al*. concluded that aerobic exercise training of moderate or high intensities in people with overweight/obesity reduces VAT more effectively than low intensity or strength training [2]. Therefore, it is plausible that an intensity threshold exists for PA to reduce VAT. However, the exact importance of PA levels for older adult population needs to be addressed in long-term studies, using preferentially relative intensities or correction for a cardiorespiratory capacity that tends to decrease with age, instead of absolute intensities (METs thresholds determined in younger adults), which are likely to underestimate time spent on MVPA [38].

Regarding PA volume, current WHO health strategies for adults indicate that engaging between 150 and 300 min of MVPA weekly is necessary for obesity management and overall health purposes. Findings from this study extend this knowledge, demonstrating that meeting these recommendations offer additional benefit for positive VAT outcomes. Our results corroborate those from the systematic review of clinical trials which showed that as little as 10 MET h/week (150 min/week) of moderate PA is adequate for VAT reduction [39]. Increasing MVPA time over 300 min/week was associated with further reduction in VAT although of lower magnitude. In line with our study, other authors observed a dose-response effect for greater intra-abdominal fat loss with increasing duration of exercise interventions [5,34].

In general, SB is associated with detrimental health outcomes and minimizing SB, as a public health strategy has been proposed. Our study provides new findings suggesting that SB may have an association with VAT, independently of PA and other potential confounders. This supports previous findings from other cross-sectional accelerometry-based studies which targeted older individuals with a high risk of chronic diseases and metabolic dysfunction [3,8], but not with studies on younger or healthier subjects [4,40]. Furthermore, we found that time spent on TV-viewing was not significantly associated with VAT. Similarly, Wilmot *et al*. concluded in a meta-analysis that TV-viewing may be a poor measure of overall SB, underestimating the true effect of overall sitting-related SB on health outcomes [41]. Undoubtedly, further research is required to better understand the associations among different domains of SB and adiposity.

In addition, when SB was categorized, we observed that the greatest benefits on VAT reduction were observed when time on SB was lower than 4 h/d. This threshold has been associated to strongest reduction in obesity (including its central type) [42] and overall mortality in previous studies [31,40]. Furthermore, analyses on the joint association between PA and SB demonstrated that the strongest effect estimate was detected when low sedentarism (<4 h/d) was accompanied of 150–300 min/week of MVPA, with a reduction of nearly 0.5 kg of VAT, compared to those that were sedentary and engaged in little or no MVPA. The combination of longer time spent on SB, and particularly watching TV, and not meeting MVPA recommendations has been previously associated with greater risk for obesity and T2D [10], increases in WC [43] and with highest amount of computed tomography-measured VAT [35]. Thus, although we found SB and PA independently associated with VAT, it seems that their relationship may be additive. This emphasizes that making recommendations related to VAT deposition should encourage both reducing sedentary time, and increasing PA.

Total body fat was not included to the minimally sufficient adjustment set in our DAG, given that this variable may act as a mediator of the aforementioned associations. Still, we decided to adjust for it in sensitivity analyses to determine the “direct effect” of PA, SB and erMedDiet on VAT independently of total body fat. These resulted in an attenuation of the associations and loss of statistical significance. This could indicate that any effect of PA and SB on VAT will be a consequence of an overall reduction in total body fat. Or that, given that VAT constitutes a very small percentage of total fat in this population (8.4% in men and 4.9% in women) it is difficult to elucidate whether PA and SB exert specific effects on VAT beyond those on body composition. Previous studies which addressed different adipose tissue depots showed that health benefits from engaging PA were related to an overall favorable reduction in total body fat and both VAT and SAT abdominal fat components [32,34,36]. This might support, at least partly, findings from our study, and confirm the need for future studies to elucidate the mechanism underlying the association of inactivity and activity with VAT.

The potential of MedDiet as a treatment strategy to reduce central obesity has been firmly evidenced in observational studies [11,12] and in the intervention trials [13,14], with the use of surrogate measures (i.e. WC) for VAT. However, the use of anthropometry for central obesity evaluation does not differentiate between either fat and lean mass or VAT and subcutaneous adipose tissue within the abdomen. Among very sparse studies that addressed to investigate aforementioned association with precise imaging-defined measures, as ultrasonography [44] or magnetic resonance imaging [45], an inverse relation between MedDiet and VAT was confirmed. In turn, in our analysis we could not support findings from the previous authors, as the association did not reach statistical significance, albeit the same tendency to decline DXA-derived VAT was observed among those participants with better adherence to an energy-restricted MedDiet. In a sensitivity analyses, the use of the widely applied MDS was only borderline significantly associated to VAT in crude models only, but lost statistical significance after adjusting for PA and other variables. These null findings could indicate that the Mediterranean diet is not specifically associated to this fat depot in obese participants; or could be a result of bias prevalent in cross-sectional studies, such as missreporting of diet in the obese or reverse causation. For the future considerations, it would be of interest to evaluate the effect of MedDiet intervention on VAT at the prospective level within the PREDIMED-Plus trial.

A marked strength of this study was the use of large, and at the same time, homogeneous sample of men and women within narrow ranges of body mass index (BMI), age and metabolic health profile. Another strong point was the use of DXA imaging technique with CoreScan automated algorithms to determine VAT amount, which is a reliable and valid method for this fat depot estimation. Furthermore, to avoid confounding for the associations of interest, the analyses were controlled for a number of factors, which were selected with use of validated and objective DAG tool within DAGitty software. Finally, additional analyses were performed showing that obtained results are not modifiable by sex, age and diabetes status.

As far as the limitations are concerned, the cross-sectional design was the major restriction that impedes inference regarding causality of the associations found. Selection of older subjects with overweight/obesity and MetS for the study cohort limits extrapolation of findings to other populations, as younger, leaner or healthier subjects, and stands for reverse causation bias. Furthermore, the present study used self-reported questionnaires to obtain data on PA, SB and erMedDiet score, which might be subject to potential biases, even though the questionnaires were validated. In order to support the self-reported data from PA and SB questionnaires, data derived from the chair-stand test, as a proxy indicator for physical fitness and lower-limb muscle strength in elderly, was included. Moreover, MVPA activity levels used in our analysis might have been underestimated, as we did not have taken into account or adjusted for person´s functional mobility, as well as cardiorespiratory or aerobic capacity that decreases with age. Lastly, our study was limited to single-race (Caucasians), hence the associations found may not be applicable to another ethnic/racial group. Due to the racial heterogeneity in lifestyle habits and fat distribution within different depots, it would be of interest to replicate our findings in diverse populations and further confirm longitudinally the present observations.

## Conclusions

In conclusion, results from this cross-sectional study revealed that in older subjects with overweigh/obesity and MetS, total and moderate-to-vigorous PA (but not light PA) is associated inversely, and total time spent on SB positively with VAT depot. In a broader context, our findings give valuable clue for clinical practice and research on which lifestyle-based methods are effective in combating morbimortality associated with obesity in this particular group of subjects. However, more studies are needed to confirm and broaden obtained data. Fortunately, since PREDIMED-Plus is an ongoing clinical trial, it will allow to extend the associations addressed here in the future longitudinal and/or interventional analysis.

## Supporting information

S1 Text List of PREDIMED-Plus investigators. (DOCX)

S1 Fig Flow chart of PREDIMED-Plus participants included in the present study. (DOCX)

S2 Fig Directed acyclic graphs (DAGs). (DOCX)

S1 Table Energy-restricted Mediterranean diet used in the intervention arm of the PREDIMED-Plus trial. (DOCX)

S2 Table Baseline characteristics of study sample by recruiting center. (DOCX)

S3 Table Additional sensitivity analysis—Further adjustment for total body fat. (DOCX)
