# Correction: Lifestyle factors and visceral adipose tissue: Results from the PREDIMED-PLUS study

**DOI:** 10.1371/journal.pone.0214837

**Published:** 2019-03-28

**Authors:** The PLOS ONE Staff

There are errors in the Author Contributions. The publisher apologizes for the errors. The correct contributions are: Conceptualization: AMG-P, JK, DR. Data curation: AMG-P, JK, IA, AC, NR-E, MAZ, ZV, RE, JVi, ET, NB, MF, RC, JVe, PB-C, JA de Paz, AG, JS-S, JAM, DR. Formal analysis: AMG-P, JK, DR. Funding acquisition: AMG-P, JK, IA, AC, NR-E, MAZ, ZV, RE, JVi, ET, NB, MF, RC, JVe, PB-C, JA de Paz, AG, JS-S, JAM, DR. Investigation: AMG-P, JK, IA, AC, NR-E, MAZ, ZV, RE, JVi, ET, NB, MF, RC, JVe, PB-C, JA de Paz, AG, JS-S, JAM, DR. Methodology: AMG-P, JK, DR. Project administration: RE, JS-S. Resources: AMG-P, JK, IA, AC, NR-E, MAZ, ZV, RE, JVi, ET, NB, MF, RC, JVe, PB-C, JA de Paz, AG, JS-S, JAM, DR. Supervision: JK, DR. Validation: JK, DR. Visualization: AMG-P, JK, IA, AC, NR-E, MAZ, ZV, RE, JVi, ET, NB, MF, RC, JVe, PB-C, JA de Paz, AG, JS-S, JAM, DR. Writing–original draft: AMG-P, JK, DR. Writing–review & editing: AMG-P, JK, IA, AC, NR-E, MAZ, ZV, RE, JVi, ET, NB, MF, RC, JVe, PB-C, JA de Paz, AG, JS-S, JAM, DR.
