# Supplementary material for: Lifestyle factors and visceral adipose tissue: Results from the PREDIMED-PLUS study
Source: PLoS One. 2019 Jan 25;14(1):e0210726. doi: 10.1371/journal.pone.0210726 (PMC6347417; doi:10.1371/journal.pone.0210726)
Supplement: S1 Text — (DOCX) [file pone.0210726.s001.docx]

S1 Text. **List of PREDIMED-Plus Investigators**

**Hospital Son Espases (HUSE) and Instituto de Investigación Sanitaria Illes Balears (IdISBa), Palma de Mallorca, Spain:** Manuel Moñino, Margarita Morey, Verónica Pereira, Maria Ángeles Martín, Aina Yáñez, Joan Llobera, Joana Ripoll, Rafael Prieto, Felix Grases, Antonia Costa, Carlos Fernández-Palomeque, Elena Fortuny, Marta Noris, Susana Munuera, Francisco Tomás, Francisca Fiol, Antoni Jover, Juana Mª Janer, Catalina Vallespir, Isabel Mattei, Natalia Feuerbach, Maria del Mar Sureda, Silvia Vega, Lourdes Quintana, Aina Fiol, Miriam Amador, Susana González, Joan Coll, Ana Moyá, Teresa Piqué y Maria Dolores Sanmartín, Cristina Peña.

**Department of Nutrition, Food Sciences, and Physiology, Center for Nutrition Research, University of Navarra, Pamplona, Spain**: Irene Cantero, Carmen Cristobo, Idoia Ibero-Baraibar, Javier Ágreda Martínez, Mª Dolores Lezáun Burgui, Nuria Goñi Ruiz, Rafael Bartolomé Resano, Eugenia Cano Cáceres, Teresa Elcarte López, Elena Echarte Osacain, Beatriz Pérez Sanz, Itziar Blanco Platero, Simón Antonio Andueza Azcárate, Álvaro Gimeno. Aznar, Eugenia Ursúa Sesma, Benito Ojeda Bilbao, Javier Martinez Jarauta, Lourdes Ugalde Sarasa, Blanca Rípodas Echarte, Mª Victoria Güeto Rubio.

**Human Nutrition Unit, University Hospital of Sant Joan de Reus, Department of Biochemistry and Biotechnology, Pere Virgili Institute for Health Research, Rovira i Virgili University, Reus, Spain:** Andres Díaz-Lopez, Roser Pedret Llaberia, Rosi Gonzalez, Ramon Sagarra Álamo, Fransec París Palleja, Josep Balsells, Josep M. Roca, Teresa Basora Gallisa, Jesús Vizcaino, Pilar Llobet Alpizarte, Carmen Anguera Perpiñá, Montse Llauradó Vernet, Carmen Caballero, Maite Garcia Barco, Maria Dolores Morán Martínez, Josep García Rosselló, Albert Del Pozo, Carmina Poblet Calaf, Pierre Arcelin Zabal, Xavier Floresví, Marta Ciutat Benet, Antonio Palau Galindo, Josep J. Cabré Vila, Fernando Dolz Andrés, Marc Soler, Mayte Gracia Vidal, J. Vilalta Juan Boj Casajuana, Maritxell. Ricard, F. Saiz, Anna Isach, M. Sánchez Marín Martinez, Esther granado Font, Carmen Lucena Luque, Mónica Bulló, Nerea Becerra-Tomás, Gloria Mestres, Josep Basora, Guilermo Mena-Sánchez, Laura Barrubés Piñol, Marta Gil Segura, Christopher Papandreou, Samira Chig, I. Abellán Cano, Veronica Ruíz García, Albert Salas-Huetos, Indira Paz, Laura Sánchez Niembro, Pablo Hernandez, Silvia Canudas, Lucia Camacho-Barcia, Jesús García-Gavilán.

**Department of Preventive Medicine and Public Health, University of Navarra-Navarra Institute for Health Research and Primary Care Centres, Pamplona, Spain:** Miguel A. Martinez-Gonzalez, Miguel Ruiz-Canela, Cristina Razquin, Maira Bes-Rastrollo, Ana Sanchez Tainta, Beatriz SanJulian Aranguren, Estibaliz Goñi, Irene Barrientos, Maria Canales, Anais Rico, Javier Basterra Gortari, Ana Garcia Arellano, Javier Diez-Espino, Oscar Lecea Juarez, Juan Carlos Cenoz Osinaga, Javier Bartolome Resano, Ana Sola-Larraza, Elisa Lozano-Oloriz, Begoña Cano-Valles, Sonia Eguaras, Elena Pascual Roquet-Jalmar, Iñigo Galilea-Zabalza, Hana Lancova, Raul Ramallal, Mª Luisa Garcia-Perez, Vicente Estremera-Urabayen, Mª Jose Ariz-Arnedo, Carmen Hijos-Larraz, Cristina Fernandez Alfaro, Begoña Iñigo-Martinez, Ramon Villanueva Moreno, Sonia Martin-Almendros, Luisa Barandiaran-Bengoetxea, Carmen Fuertes-Goñi, Ana Lezaun-Indurain, Mª Jose Guruchaga-Arcelus, Oscar Olmedo-Cruz, Begoña Iñigo-Martínez, Luis Escriche-Erviti, Roberto Ansorena-Ros, Rosario Sanmatin Zabaleta, Jon Apalategi-Lasa, Jerusalen Villanueva-Telleria, Mª Mar Hernández-Espinosa, Isabel Arroyo-Bergera, Lissy Herrera-Valdez, Lourdes Dorronsoro-Dorronsoro.

**Department of Endocrinology and Nutrition, Hospital Clínic, Barcelona, Spain:** Carla Mestre Reina, Emilio Ortega Martínez de la Victoria, Irene Vinagre Torres, Judit Viaplana Masclans, José Ignacio Pla Puig, Tania-Marisa, Freitas-Simoes.

**Department of Internal Medicine, Hospital Clínic, IDIBAPS August Pi i Sunyer Biomedical Research Institute, University of Barcelona, Barcelona, Spain:** M. Domenech, C. Sierra, M. Camafort, Gabriel Fontana, S. Castro, M. Sadurni, C. Viñas, P. Villanueva, R. Soriano, M. de la Poza, J.M. Cots, J. M. Llovet, C. Carbonell, Y. García, J. Altirriba, V. Aragunde.

**Grupo de Investigación en Interacciones Gen-Ambiente y Salud, Universidad de León y Gerencia de Atención Primaria. Sacyl, León, Spain:** Tania Fernández Villa, Serafín de Abajo Olea, Miguel Escobar Fernández, Jose Pedro Fernández Vázquez, Jaime López de la Iglesia, Juan Ignacio López Gil, Elena Carriedo Ule, Manuel Rodríguez Bul, Abdurrahman Adlbi Sibai, Alberto García Hernández.
