# Supplementary material for: Lifestyle factors and visceral adipose tissue: Results from the PREDIMED-PLUS study
Source: PLoS One. 2019 Jan 25;14(1):e0210726. doi: 10.1371/journal.pone.0210726 (PMC6347417; doi:10.1371/journal.pone.0210726)
Supplement: S1 Fig — (DOCX) [file pone.0210726.s002.docx]

S1 Fig. **Flow chart of PREDIMED-Plus participants included in the present study.**

Abbreviations: DXA - dual-energy X-ray absorptiometry; VAT - Visceral adipose tissue
