# Supplementary material for: Lifestyle factors and visceral adipose tissue: Results from the PREDIMED-PLUS study
Source: PLoS One. 2019 Jan 25;14(1):e0210726. doi: 10.1371/journal.pone.0210726 (PMC6347417; doi:10.1371/journal.pone.0210726)
Supplement: S2 Fig — (DOCX) [file pone.0210726.s003.docx]

S2 Fig. **Directed acyclic graphs (DAGs).**

A

**
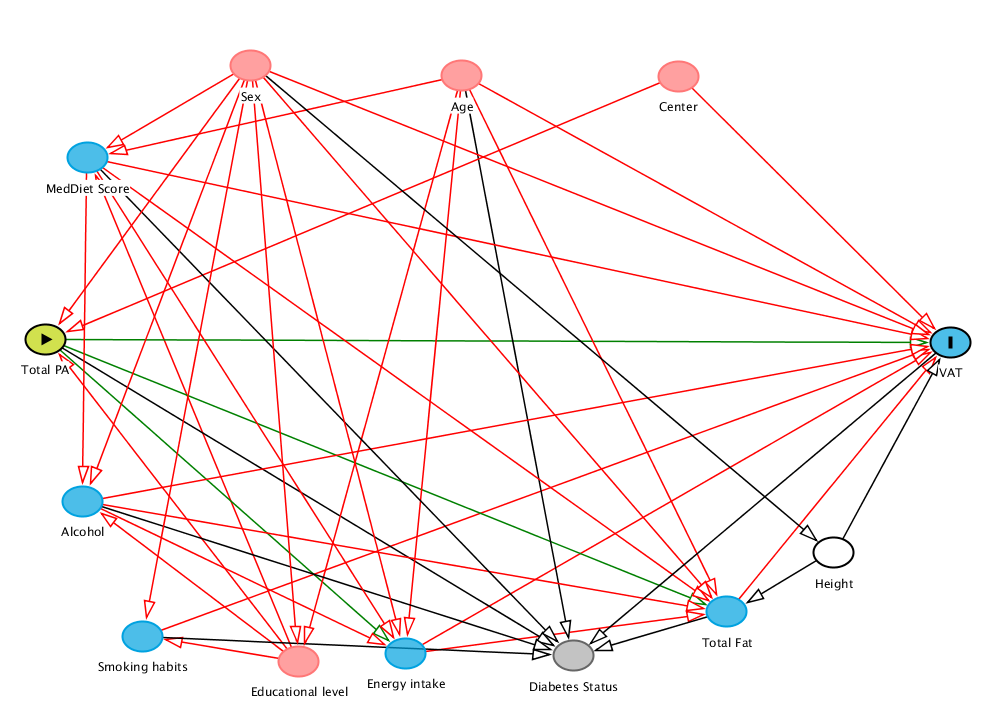
**

B


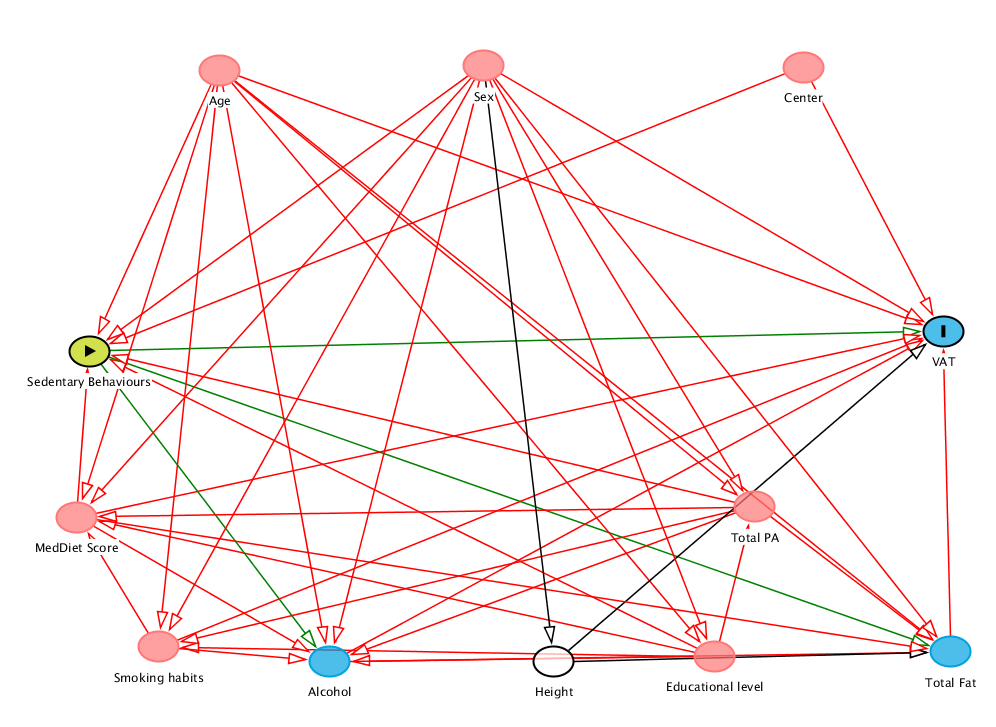


**C**


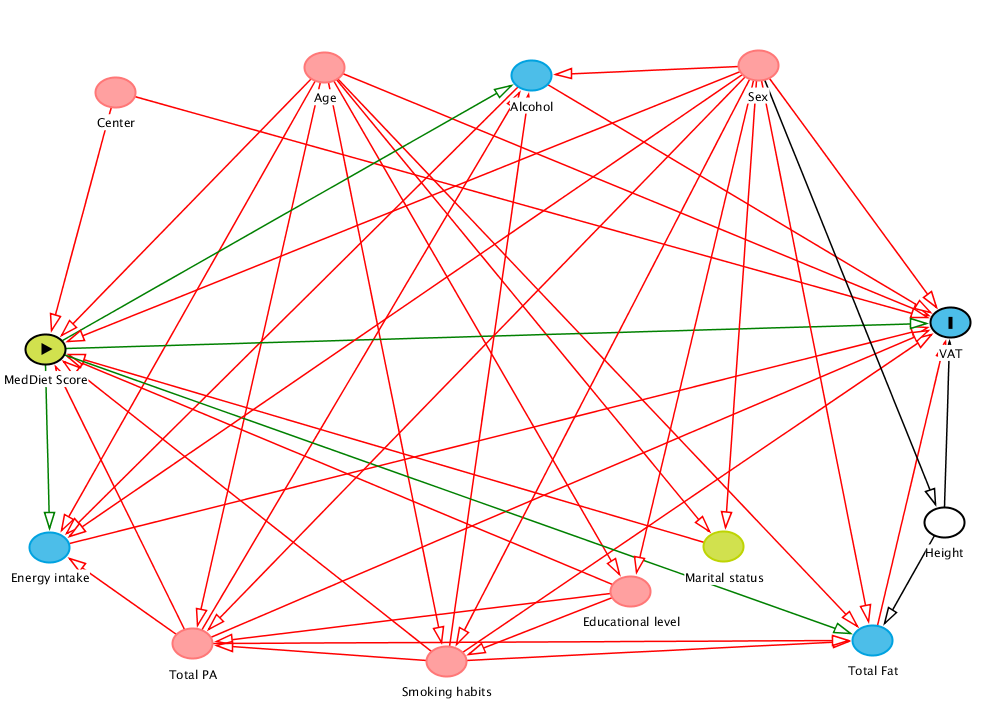


**Figure legend:**

**Additional file: Figure 1. Directed acyclic graphs (DAGs).** The total unconfounded effect of total PA (A), total SB (B), and erMedDiet score (C) on VAT, drawn and analyzed using DAGitty ([www.dagitty.net](http://www.dagitty.net)). The minimally sufficient adjustment set (MSAS) for total PA (A) was age, alcohol, center, energy intake, height, erMedDiet score, sex, total fat and educational level and smoking habits; the MSAS for total SB (B) was age, alcohol, center, height, erMedDiet score, sex, smoking habits, total fat; and the MSAS for erMedDiet score was age, alcohol, center, energy intake, height, sex, smoking habits, total fat, and total PA.

Abbreviations: PA – physical activity, SB – sedentary behaviours, VAT – visceral adipose tissue, erMedDiet – energy-restricted Mediterranean diet.
