# Supplementary material for: Lifestyle factors and visceral adipose tissue: Results from the PREDIMED-PLUS study
Source: PLoS One. 2019 Jan 25;14(1):e0210726. doi: 10.1371/journal.pone.0210726 (PMC6347417; doi:10.1371/journal.pone.0210726)
Supplement: S1 Table — (DOCX) [file pone.0210726.s004.docx]

S1 Table. **Energy-restricted Mediterranean diet used in the intervention arm of the PREDIMED-Plus trial**.

17-point questionnaire to assess adherence to the energy-restricted Mediterranean diet.

| **Questions** | **Criteria for 1 point** |
| --- | --- |
| 1. Do you use only extra-virgin olive oil for cooking, salad dressings, and spreads? | Yes |
| 1. How many fruit units (including natural fruit juices) do you consume per day? | ≥3 |
| 1. How many servings of vegetables/garden produce do you consume per day? [1 serving: 200 g (consider side dishes as half a serving)] | ≥2 (≥1 portion raw or in a salad) |
| 1. How many servings of white bread do you consume per day? (1 serving: 75 g) | ≤1 |
| 1. How many times per week do you consume whole grain cereals and pasta? | ≥5 |
| 1. How many servings of red meat, hamburgers, or meat products (ham, sausage, etc.) do you consume per week? (1 serving: 100-150 g) | ≤1 |
| 1. How many servings of butter, margarine, or cream do you consume per week? (1 serving: 12 g) | <1 |
| 1. How many sugary beverages or sugar-sweetened fruit juices do you drink per week? | <1 |
| 1. How many servings of legumes do you consume per week? (1 serving: 150 g) | ≥3 |
| 1. How many servings of fish or shellfish do you consume per week? (1 serving: 100-150 g of fish or 4-5 units or 200 g of shellfish) | ≥3 |
| 1. How many times per week do you consume commercial sweets or pastries (not homemade), such as cakes, cookies, sponge cake, or custard? | <3 |
| 1. How many servings of nuts (including peanuts) do you consume per week? (1 serving: 30 g) | ≥3 |
| 1. Do you preferentially consume chicken, turkey or rabbit instead of beef, pork hamburgers or sausages? | Yes |
| 1. How many times per week do you consume vegetables, pasta, rice or other dishes seasoned with *sofrito* (sauce made with tomato and onion, leek or garlic and simmered in olive oil)? | ≥2 |
| 1. Do you preferentially add non-caloric artificial sweeteners to beverages (such as coffee or tea) instead of sugar? | Yes |
| 1. How many times per week do you consume non-whole grain pasta or white rice? | <3 |
| 1. How many glasses of wine do you drink per day? (1 glass: 200 ml) | 2-3 for men  1-2 for women |
