# Supplementary material for: Lifestyle factors and visceral adipose tissue: Results from the PREDIMED-PLUS study
Source: PLoS One. 2019 Jan 25;14(1):e0210726. doi: 10.1371/journal.pone.0210726 (PMC6347417; doi:10.1371/journal.pone.0210726)
Supplement: S2 Table — (DOCX) [file pone.0210726.s005.docx]

S2 Table. **Baseline characteristics of study sample by recruiting center.**

| n=1231 | Center A | Center B | Center C | Center D | Center E | Center F |  |
| --- | --- | --- | --- | --- | --- | --- | --- |
|  | n=241 | n=298 | n=142 | n=336 | n=99 | n=115 | *p-value* |
| Age (years) | 65.7 (5.1) | 65.0 (4.8) | 66.0 (4.8) | 64.9 (5.1) | 63.8 (4.9) | 66.9 (5.0) | **<0.001** |
| Sex: |  |  |  |  |  |  | 0.277 |
| Men | 128 (53) | 152 (51) | 74 (52) | 178 (53) | 62 (63) | 53 (46) |  |
| Women | 113 (47) | 146 (49) | 68 (48) | 158 (47) | 37 (37) | 62 (54) |  |
| BMI (kg/m^2^) | 32.1 (3.2) | 32.8 (3.3) | 32.6 (3.1) | 33.0 (3.4) | 31.8 (3.2) | 32.1 (3.5) | **0.001** |
| Height (cm) | 164 (9) | 162 (9) | 163 (9) | 162 (9) | 168 (9) | 162 (9) | **<0.001** |
| VAT (kg) | 2.38 (0.88) | 2.23 (0.83) | 2.50 (1.10) | 2.10 (0.80) | 2.53 (1.00) | 2.36 (0.85) | **<0.001** |
| Total fat (kg) | 33.8 (7.2) | 35.1 (6.9) | 35.7 (7.1) | 33.8 (7.3) | 34.3 (6.7) | 34.6 (7.6) | **0.039** |
| MetS components prevalence: |  |  |  |  |  |  |  |
| Abdominal obesity | 229 (95) | 291 (98) | 137 (96) | 322 (96) | 95 (96) | 113 (98) | 0.520 |
| Hyperglycemia | 187 (78) | 231 (78) | 96 (68) | 231 (69) | 77 (78) | 96 (83) | **0.004** |
| Hypertriglyceridemia | 120 (50) | 165 (55) | 76 (53) | 192 (57) | 56 (57) | 66 (57) | 0.585 |
| Low HDL-cholesterol | 120 (50) | 151 (51) | 57 (40) | 143 (43) | 45 (45) | 48 (42) | 0.135 |
| Hypertension | 233 (97) | 264 (89) | 133 (94) | 320 (95) | 96 (97) | 99 (86) | **<0.001** |
| Total PA (MET·min/day) | 404 (337) | 413 (358) | 443 (312) | 358 (341) | 406 (370) | 384 (270) | 0.150 |
| MVPA (MET·min/day) | 304 (333) | 312 (344) | 289 (304) | 242 (307) | 339 (361) | 219 (246) | **0.006** |
| Light PA (MET·min/day) | 100 (115) | 101 (122) | 153 (159) | 116 (139) | 66.5 (102) | 165 (168) | **<0.001** |
| Chair-stand test (repeats) | 13.5 (4.6) | 15.4 (5.0) | 17.5 (7.4) | 11.2 (3.5) | 14.7 (4.7) | 14.0 (4.9) | **<0.001** |
| Total SB (h/day) | 5.84 (1.88) | 6.00 (1.63) | 5.32 (1.98) | 5.93 (1.77) | 4.87 (1.69) | 6.20 (1.78) | **<0.001** |
| TV-viewing SB (h/day) | 3.09 (1.62) | 3.58 (1.67) | 2.95 (1.48) | 2.87 (1.50) | 2.49 (1.30) | 3.29 (1.91) | **<0.001** |
| erMedDiet score (points) | 8.98 (2.58) | 8.18 (2.79) | 7.31 (2.58) | 7.96 (2.36) | 9.29 (2.77) | 8.70 (2.41) | **<0.001** |
| MDS (points) | 4.70 (1.62) | 3.56 (1.65) | 3.58 (1.82) | 4.20 (1.51) | 4.22 (1.51) | 4.53 (1.55) | **<0.001** |
| Alcohol (g/day) | 11.7 (16.4) | 9.69 (14.0) | 11.3 (14.5) | 11.2 (13.8) | 15.1 (18.7) | 12.0 (13.6) | 0.066 |
| Smoking habits: |  |  |  |  |  |  | 0.077 |
| Never | 93 (39) | 118 (40) | 62 (44) | 157 (47) | 35 (35) | 62 (54) |  |
| Current | 37 (15) | 35 (12) | 15 (11) | 33 (10) | 12 (12) | 14 (12) |  |
| Former | 111 (46) | 145 (49) | 65 (46) | 146 (43) | 52 (53) | 39 (34) |  |
| Educational level: |  |  |  |  |  |  | **<0.001** |
| Higher education/technician | 63 (26) | 51 (17) | 44 (31) | 52 (15) | 24 (24) | 34 (30) |  |
| Secondary education | 60 (25) | 77 (26) | 44 (31) | 111 (33) | 32 (32) | 44 (38) |  |
| Primary education/illiterate | 118 (49) | 170 (57) | 54 (38) | 173 (51) | 43 (43) | 37 (32) |  |

Data are expressed as means and standard deviations (SDs) for continuous variables or numbers and percentages (%) for categorical variables. *P*-value threshold was set at <0.05 and determined using one-way analysis of variance (ANOVA) for continuous variables and chi-square test (χ²) for categorical variables.

MetS components, as one of the major inclusion criteria were evaluated at the first screening visit during run-in period, according to the harmonized definition of the joint statement from the International Diabetes Federation/National Heart, Lung and Blood Institute/American Heart Association (2009) [1].

Abbreviations: erMedDiet – energy-restricted Mediterranean diet; MetS – metabolic syndrome; PA – physical activity; MVPA – moderate-to-vigorous physical activity; SB – sedentary behaviours, VAT – visceral adipose tissue.

**References**

1. Alberti KGMM, Eckel RH, Grundy SM, Zimmet PZ, Cleeman JI, Donato KA, et al. Harmonizing the metabolic syndrome: a joint interim statement of the International Diabetes Federation Task Force on Epidemiology and Prevention; National Heart, Lung, and Blood Institute; American Heart Association; World Heart Federation; International Atherosclerosis Society; and International Association for the Study of Obesity. Circulation. 2009;120: 1640–1645. doi:10.1161/CIRCULATIONAHA.109.192644
