# Supplementary material for: Lifestyle factors and visceral adipose tissue: Results from the PREDIMED-PLUS study
Source: PLoS One. 2019 Jan 25;14(1):e0210726. doi: 10.1371/journal.pone.0210726 (PMC6347417; doi:10.1371/journal.pone.0210726)
Supplement: S3 Table — (DOCX) [file pone.0210726.s006.docx]

S3 Table. **Additional sensitivity analysis – further adjustment for total body fat.**

| (n = 1231) | β | (95% CI) | *p-value* |
| --- | --- | --- | --- |
| Total PA (100 MET·min/day) | -3.13 | (-13.3; 7.0) | 0.546 |
| Light PA (100 MET·min/day) | 15.3 | (-9.32; 40.0) | 0.223 |
| MVPA (100 MET·min/day) | -6.33 | (-17.0; 4.35) | 0.245 |
| Chair-stand test (repeats) | 3.75 | (-3.30; 10.8) | 0.297 |
| TV-viewing SB (h/day) | -6.93 | (29.1; 15.2) | 0.540 |
| Total SB (h/day) | -2.34 | (-21.7; 17.0) | 0.813 |
| erMedDiet score (points) | -4.18 | (-17.5; 9.11) | 0.537 |

Adjustment for total body fat was performed to estimate the direct effect of our exposures on VAT (taking into account plausible mediators, according to our DAG). The associations were estimated using linear regression models adjusted for age, sex, center, height, total body fat, and erMedDiet score (for models with exposures total PA, light PA, MVPA, chair-stand test, total SB and TV-viewing SB), alcohol (total PA, light PA, MVPA, chair-stand test), smoking habits (total PA, light PA, MVPA, chair-stand test, erMedDiet score), educational level (total SB and TV-viewing SB), and total PA (erMedDiet score).
